# What are the sleep characteristics among early kidney transplant recipients? An objective and subjective measurement from China

**DOI:** 10.1371/journal.pone.0277678

**Published:** 2022-11-22

**Authors:** Zhihao Zhang, Jia Liu, XiaoXia Wu, Jin Yan

**Affiliations:** 1 School of Public Administration, Central South University, Changsha, Hunan, China; 2 Xiangya Nursing School, Central South University, Changsha, Hunan, China; 3 Department of Nursing, The Third Xiangya Hospital of Central South University, Changsha, Hunan, China; 4 Transplantation Center, The Third Xiangya Hospital of Central South University, Changsha, China; UNITED STATES

## Abstract

**Objective:**

To evaluate the sleep quality by self-reported questionnaires and polysomnography (PSG) among early kidney transplant recipients (KTRs) and to further explore their correlation.

**Design:**

This was a prospective and cross-sectional analysis of the sleep characteristics among early kidney transplant recipients through an objective and subjective measurement.

**Participants:**

Patients with end stage renal disease on the transplant waiting list and after kidney transplantation were from a major organ transplantation center in Southern China (n = 83).

**Primary and secondary outcome measurements:**

Objective outcomes: PSG, noise and light. Subjective outcomes: demographic and clinical questionnaires, self-reported pain and Richards Campbell sleep questionnaire (RCSQ). After agreement with the informed consent, participants first completed demographic and clinical questionnaires, then worn the PSG within 5–10 days after kidney transplantation. Both noise, light and self-reported pain were monitored during sleep. After completion of PSG, the RCSQs were filled out next morning.

**Results:**

A total of 298 patients were recruited and 83 participants were finally analyzed. The total RCSQ mean score was 51.0±18.9mm. The prevalence of poor sleep quality among early KTRs was 45.1%. Most of PSG characteristics were significantly correlated with their corresponding RCSQ items. And the total RCSQ scores were significantly correlated with the number of awakenings, the N2 percentage and the total sleep time (r = 0.79, 0.47 and 0.40, P<0.05) respectively. Noise was a statistically significant factor affecting the subjective sleep quality.

**Conclusions:**

The sleep quality in early KTRs measured by both PSG and RCSQ exhibits consistency with each other. Sleep disruption always remains a substantial problem and is affected by self-reported noise among early KTRs. The RCSQ is easily applicable and interpretable so that it can be used for future daily clinical practice.

## 1. Introduction

Sleep disorders are the most common comorbidities in patients with end stage renal disease (ERDS). They could cause abnormal sleep behaviors and poor sleep quality, which generate some undesirable health consequences, such as fatigue [1], depression [2], decreased quality of life [3], premature mortality [4] as well as major health economic consequences [5].

Kidney transplant (KT) is by far the most desirable treatment for patients with ERDS. It seems that successful KT could restore normal metabolism and has the ability to improve sleep quality [6]. Our previous research [7] found that about 29.2% kidney transplant recipients (KTRs) have experienced poor sleep (defining as global Pittsburgh sleep quality index—PSQI>7) during follow-up monitoring (64% post KT period >one year). Other previous studies [8, 9] revealed that 24.5% ~ 62% of KTRs show poor sleep quality and persist this problem within one year after KT. Sleep disorders in KTRs may occur as a result of having only one kidney, using hormone and immunosuppressant as well as other comorbidities (e.g. high risk of cardiovascular disease, malignancies, and anxiety upon loss of allograft) [10]. These will cause functional decline in patients’ daytime life, with increased fatigue, excessive daytime sleepiness and may also have negative effects on their behavior, cognition and attention [1, 11].

Poor sleep could be defined using specific diagnostic criteria subjectively and objectively. Commonly used questionnaires include Richards Campbell sleep questionnaire (RCSQ) [12], Epworth Sleepiness Scale [13], and the Berlin Questionnaire [14]. These subjective questionnaires are simple and convenient methods for preliminary sleep disorder screen and diagnosis. However, all these self-reported questionnaires only provide general described or categorized results on sleep symptoms but lack further physiological analysis of sleep. Polysomnography (PSG) is a powerful tool for diagnosing sleep-related diseases and sleep research. It provides quantitative and qualitative information of sleep by analyzing sleep physiological parameters. Although PSG intuitively reflects the patients’ sleep quality at night and is considered as the gold standard for diagnosing and evaluating some sleep-related illness, only a few published articles [15–18] reported the use of PSG in KTRs because of its inconvenience in wearing.

Therefore, the main purpose of this study is to evaluate the sleep quality by self-reported questionnaires and PSG among early KTRs and to further explore their correlation. Additionally, this study is also aim to analyze possible risk factors related to the self-reported sleep quality.

## 2. Methods

### 2.1 Design, setting and participants

This was a prospective and cross-sectional study using convenient sampling. All KTRs who admitted in the Third Xiangya Hospital of Central South University in Hunan Province, China from August 2018 to March 2021 were recruited (Information about transplant surgery, S1 Appendix). The inclusion criterias were (1) Age 18 to 60 years old; (2) All body indexes were in a stable condition after kidney transplantation. And PSGs were acceptable to participants after evaluating by physician; (3) No physical or mental conditions were involved in to prevent the participants from completing the survey and monitoring by PSG. The exclusion criterias were (1) Individuals took medications with known effects on sleep physiology such as antihistamines, antidepressants, and anticonvulsants or with major mental illness required psychiatric treatment. Taking corticosteroids was not included in the exclusion criterias as it’s an important part of immunosuppressive regimen; (2) Previously diagnosed and/or treated for sleep disorders such as sleep apnea (SA), periodic limb movement disorder (PLMD); (3) Failure in PSG monitoring and/or incomplete sleep data.

Each donors of KTRs were from donation after cardiac death (DCD) according to the standard organ donation registration and distribution system made by Chinese government (www.cot.org.cn).

The Ethical approval was obtained from the Institutional Review Board (IRB) of the Third Xiangya Hospital of Central South University (No:2017-S222). All KTRs signed the informed consent at the beginning of this study. Every KTRs received an open published book of “Essentials you need to know after kidney transplant” (12 RMB) or a medicine container (15 RMB) as a compensation for spending their time.

### 2.2 Variables and measurement

**Fig 1:**
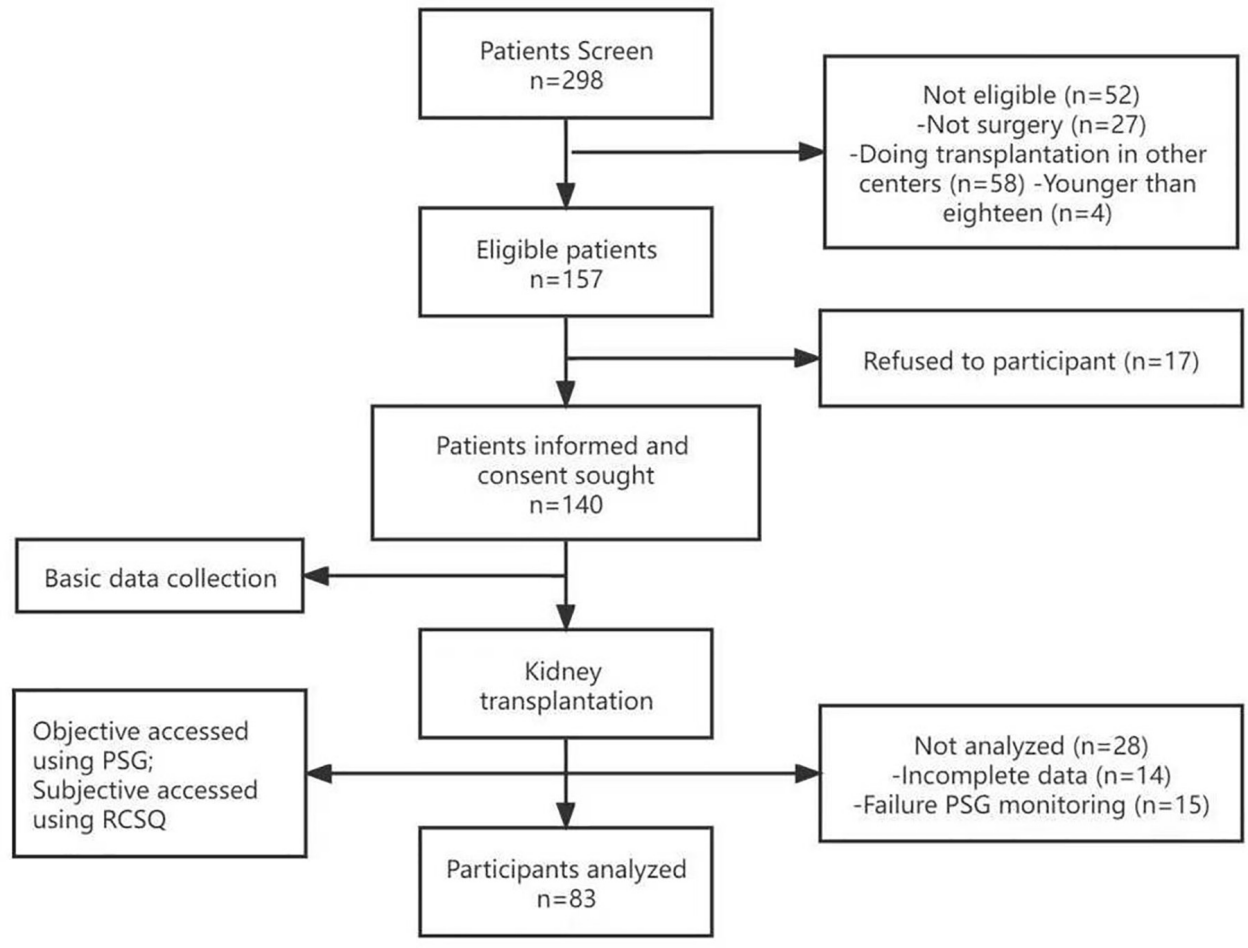
Flowchart of case collection.

A research assistant approached eligible patients prior to transplant operation. If they were interested, they were invited to participate in a targeted assessment with informed consent. Then they completed baseline assessments of demographics through self-reported questionnaires within 5–10 days after transplantation when their vital signs were in a stable condition. The variables of sleep contained self-reported questionnaires (RCSQ) and polysomnography (PSG). The PSG was worn by trained technicians (two consecutive nights and the data from the first night was discarded) and the subjective sleep scale (RCSQ) was completed on the third morning. The time for PSG to be worn was arranged according to the night bedtime routine of participants. Then the data were processed by trained technicians who did not know the clinical characteristics of patients. Sleep stages were scored according to guidelines developed by Rechtschaffen and Kales [19]. Meanwhile, self-reported pain degree, noises and illumination were also collected during the sleep monitoring. The overview of KTRs collection was depicted in Fig 1.

#### 2.2.1 Demographics and clinical characteristics

Demographic data such as age, gender, Body Mass Index (BMI), occupation, status of marriage or fertility, degree of education, monthly income, sleep pattern were collected by interviewing. Clinical questionnaire include primary disease, the type of dialysis before KT, dialysis duration before transplant, waiting time for transplant, frequency of transplant, types and dosages of immunosuppressant, sleep drugs or analgesic after transplant were obtained from hospital information system or interviewing.

#### 2.2.2 Objective sleep assessment- polysomnography (PSG)

The PSG (EEG polysomnography monitor Acumen7) was performed overnight for early KTRs when they were on postoperative isolation ward. This technique employs numerous collections of surface electrodes which measure physiologic parameters of sleep including brain dynamics of EEG, eye movements, muscle activities, heart physiology, and respiratory function [20]. Finally, data from the PSG were collected and the sleep characteristics were analyzed and concluded based on following parameters: sleep onset latency (SOL): The duration time between the time patient tried to sleep when the lights were turned off and the time patient actually falled asleep; wake after sleep onset (WASO): the time spent awake from sleep onset of finally awakening; total sleep time (TST): the entire time spent during sleep; times and percentages of non-rapid eye movement stages (N1, N2, N3) and latency of rapid eye movement (REM), sleep efficiency (SE). All these sleep characteristics from the PSG reflected five domains: sleep depth, difficulty in falling asleep, number of awakenings, percent of awakening time, and overall sleep quality.

#### 2.2.3 Subjective sleep assessment-Richards Campbell sleep questionnaire (RCSQ)

RCSQs were required to fill out when early KTRs with one overnight PSG. RCSQ was developed by Richards et al. [12] which consisted of five items including sleep depth, difficulty falling asleep, number of awakenings, difficulty in re-sleeping, and overall sleep quality, self-reported noise as the sixth parameter along with five items. Each item was scored using visual analogue scale from 0~100mm, higher score means better sleep. Patients marked the distance on the line represented each item score. The mean modified RCSQ score was 51.2±12.7 mm. According to the report from Naik et al., patients with RCSQ score ≤ 50 mm were considered as poor sleep [21]. A cutoff score of ≥48 mm of modified RCSQ had a sensitivity 88.2% and specificity of 73.3% (ROC area 0.88, CI: 95%) in defining patients with good sleep [12]. The Chinese version of the RCSQ scale has a content validity of 0.840 and a Cronbach’s α coefficient of 0.874 [22].

#### 2.2.4 Other variables

Visual analogue scale (VAS) was used to assess the self-reported pain degree during sleeping monitoring stage, noise, and illumination were also detected by digital sound level meter (SMART AR844) and luminometer (TES-1330A).

### 2.3 Statistical analysis

All data were reviewed by the double check. If the missing values in the questionnaire was >20% or key values (number of awakenings, total sleep time et al.) were missing in the PSG, the data in this case was invalid. Statistical analyses were performed using SPSS 25.0 (SPSS Inc., Chicago, IL). The continuous data were described as mean and standard deviation, while the categorical data were described as frequencies and percentages. Correlations between RCSQ and PSG were tested using the Spearman correlation test. Bivariate logistic regression analysis was used for exploring the related factors among sleeping parameters.

### 2.4 Patient and public involvement

Patients were involved in the design and conduct of the research. Working with patients at the bedside enabled us to identify the serious sleep problem of this population and motivated us to undertake this research endeavour. While receiving and reviewing the results together with all participants to verify the findings, the research team still seeked additional and appropriate feedback.

## 3. Results

### 3.1 Sample characteristics

298 patients were invited to participate in this study, with 157 (52.8%) eligible participates and 17 refused individuals. Consequently, 140 patients utilized PSG and RSCQ to evaluate their subjective and objective sleep characteristics. Among these 140 patients, 14 individuals showed incomplete data, 15 individuals were failure to complete PSG monitoring and 28 individuals were not analyzed. Finally, 83 participants were eligible for further analyses. The study profile including reasons lacking follow-up investigation was shown in Fig 1. The sleep characteristics of 83 examined early KTRs were summarized in Table 1, with the mean age of 43.33 (9.10) years old. The majority sex was male (65.1%) and the type of dialysis before transplantation was hemodialysis (76.5%). Most of patients received immunosuppressive medicine based on tacrolimus (77.2%). Steroids treatment was performed for all recipients during the initial post transplant, especially 8 (9.6%) patients received high dose due to some rejection reasons [23]. Noise and illumination detected in the sleep environment were *51*.5 (4.6) dB, 71.6 (31.2) Lux, respectively.

**Table 1:** Demographics and clinical characteristics of participants (n = 83).

Parameters	Mean (SD)	Number (%)
Age	43.3 (9.1)	
Sex		
Males		54 (65.1%)
Females		29 (34.9%)
Type of dialysis before transplant		
Hemodialysis		63 (76.5%)
Peritoneal dialysis		12 (13.8%)
Both of those		8 (9.7%)
Main regimens of immunosuppressant		
Based on Cyclosporine		13 (15.6)
Based on Tacrolimus		64 (77.2)
Others		6 (7.2)
Use of steroids		83 (100)
Use of analgesics		52 (62.4%)
Self-reported pain (VAS)	4.3 (3.0)	
Actual measured noise (dB)	51.5 (4.6)	
Illumination (Lux)	71.6 (31.2)	

### 3.2 Self-reported sleep quality of KTRs

The total RCSQ mean score (5 items) from participants was 51.0±18.9 mm. The prevalence of poor sleep among early KTRs was 45.1% in our research. The item 3 (53.7±25.8) and item 4 (53.7±30.3) showed highest scores, which correspond to the number of awakenings and the percentage of awakening time in sleep domains, respectively. All detailed results were present in Table 2.

**Table 2:** Score of total RCSQ and each item (n = 83).

Items	Mean (SD)	Range (min, max)
Item1 deep sleep/light sleep	45.5 (23.9)	(0, 90)
Item 2 fall asleep immediately/never could fall asleep	52.7 (32.1)	(0, 100)
Item 3 awake very little/awake all night alone	53.7 (25.8)	(0, 100)
Item 4 go back to sleep immediately/couldn’t get back to sleep	53.7 (30.2)	(0, 100)
Item 5 a good night sleep/a bad night sleep	49.4 (25.9)	(0, 100)
Total RCSQ score with 5 parameters	51.0 (18.9)	(12, 96)
Self-reported noise	45.3 (25.5)	(0, 100)
Total modified RCSQ score with noise	50.1 (13.5)	(27, 80)

### 3.3 PSG sleep characteristic and correlation with RCSQ

The sleep characteristics and results obtained by PSG were shown in Table 3. The total sleep time was 135.5 minutes, percent N2 and N3 represented the sleep depth which were 38.9% and 2.5%, respectively, and the sleep latency was 14.2 minutes. The number of awakenings was 8 times and the awakening time accounted for 44.8% of the total sleep time. Most PSG characteristics were significantly correlated with their corresponding RCSQ items, and RCSQ total scores were significantly correlated with the number of awakenings, percent N2 and the total sleep time (r = 0.79, 0.47 and 0.40, *P*<0.05).

**Table 3:** Descriptive statistics and correlation of PSG sleep characteristics with RCSQ items.

Sleep domains	PSG sleep characteristics	PSG [mean (SD)]	Corresponding RCSQ items	r
Sleep depth	Percent N2 (%)	38.9 (24.8)	Item1	-0.17
	Percent N3 (%)	2.5 (5.0)		-0.27*
Falling asleep	sleep onset latency (min)	14.2 (16.3)	Item 2	-0.21
Number of awakenings	Number of awakenings (times)	8.0 (5.4)	Item 3	-0.39**
Percent of time awake	Percent of time awake (%)	44.8 (32.7)	Item 4	-0.43**
Quality of sleep	Total sleep time (min)	135.5 (89.1)	Item 5	0.53**
	Percent N2 (%)	38.9 (24.8)		0.43**
	Latency to sleep onset (min)	14.2 (16.3)		0.35*
Overall perception of sleep	Total sleep time (min)	135.5 (89.1)	RCSQ total	0.40**
	Number of awakenings (times)	8.0 (5.4)		0.79**
	Percent N2 (%)	38.9 (24.8)		0.47*
	Percent REM (%)	0.6 (2.2)		0.19

r = Correlation between the PSG sleep characteristics and corresponding RCSQ items

*p<0.05

**p<0.01.

### 3.4 Correlation between clinical parameters with RCSQ and PSG

In Table 4, the RCSQ score of 50mm was taken as cutoff value to compare the patients with poor sleep (n = 23, 45.1%) and good sleep (n = 28, 54.9%) groups. Both the actual measured noise (dB), the self-reported noise (RCSQ), and the self-reported pain (VAS) showed statistically significance between these two groups (p = 0.04, p<0.01, p<0.01, respectively). The actual measured noise (dB) was also significantly associated with the total sleep time and the number of awakenings (r = 0.28, 0.29, *P*<0.05), while the self-reported noise was associated with sleep efficiency and the total awakening time (r = 0.54, 0.38, *P*<0.01). The self-reported pain was negatively correlated with the total sleep time and sleep efficiency but was positively correlated with the total awakening time (r = -0.52, -0.48, and 0.32, *P*<0.05), both of which were statistically significant as shown in Table 5.

**Table 4:** Univariate analysis of sleep quality based on RCSQ total score.

RCSQ Parameters	Poor sleepers (n = 37)	Good sleepers (n = 46)	*P*
	RCSQ<50mm	RCSQ≥50mm	
Actual measured noise (dB)	51.2 (6.1)	48.1 (4.4)	0.04*
Self-reported noise (RCSQ)	60.0 (23.0)	33.2 (20.9)	0.002**
Self-reported pain (VAS)	5.4 (2.2)	3.6 (1.8)	0.009**
Illumination (lux)	72.0 (40.1)	70.5 (34.2)	0.89
Use of analgesics			0.51
Yes	26	28	
No	11	18	

*p<0.05

**p<0.01.

**Table 5:** Correlation of PSG sleep characteristics with clinical parameters.

Parameters	sleep onset latency	Total sleep time	Sleep efficiency	Number of awakenings	Total awakening time
Actual measured noise (dB)	-0.14	-0.28*	-0.23	0.29*	0.13
Self-reported noise (RCSQ)	0.32	-0.26	-0.54**	0.22	0.38**
Self-reported pain (VAS)	-0.07	-0.52**	-0.48**	0.17	0.32*
Illumination (lux)	0.09	-0.03	-0.21	-0.09	0.03

r = Correlation between the PSG sleep characteristics and clinical parameters

*p<0.05

**p<0.01.

### 3.5 Bivariate logistic regression analysis of related factors affecting subjective sleep quality after transplantation

The self-reported sleep quality was taken as an dependent variable. General demographic data such as gender, BMI, sleep pattern, etc. and clinical data including dialysis time and types, dosages of immunosuppressants, self-reported pain (VAS), self reported noise, etc. were used as independent variables. Although the self-reported pain (VAS) (OR = 0.74, P = 0.07) and the self-reported noise (from RCSQ) (OR = 0.62, P<0.01) all entered the regression equation, only the self reported noise was a statistically significant factor affecting subjective sleep quality (p<0.01) as shown in Table 6.

**Table 6:** Bivariate logistic regression analysis of related factors affecting self-reported sleep quality after transplantation.

Variables	β coefficient	SE	Wald	OR	*P*
Self reported noise (RCSQ)	-0.47	0.17	7.68	0.62	0.004**
Self reported pain (VAS)	-0.31	0.17	3.19	0.74	0.07

*p<0.05

**p<0.01.

## 4. Discussion

There are growing evidences shown the importance of the sleep quality on KTRs after receiving transplant for a long stretch of time [2, 3, 6, 24]. This study characterized the sleep quality in early period among early KTRs by using subjective and objective measurements.

Our study found that there is a high prevalence of poor sleep in early KTRs whose sleep/wake cycle was fragmented as measured by PSG, with less than two and half hours’ total sleep time overnight and more than 8 awakening times. Our data is consistent with another published sleep survey where the KTRs manifest reduced REM sleep, fragmented sleep/wake cycle, increased arousal, and reduced deep sleep [25]. Meanwhile, the retrospective and objective RCSQ score measured from next morning showed that almost half early KTRs (23/51, 45.1%) are poor sleepers. The mean RCSQ score (51.0 ± 18.9 mm) was lower than the mean score obtained (60 ± 27 mm) from Richard’s original validation study from 70 male ICU patients [12]. Various publications have reported that medications such as non-steroidal anti-inflammatory drugs and immunosuppressants could affect sleep [26–28]. So the early KTRs went through higher doses of these drugs than the KTRs from follow-up stage. Moreover, previous reported potential factors have been shown to cause sleep disorders including transplant surgery, hospitalization, anxiety and uncertainty, fear of organ rejection, and psychosocial problems in KTRs [29]. These sleep disorders were also observed in early KTRs. Notably, another study from 104 postoperative patients showed that the mean RCSQ in the first postoperative night was 51.42 mm [30] which is similar to our findings. It is well-known that indwelling catheters and sound pain are the two major factors affecting early postoperative participants. All of these might support the fact that why the participants in our research experienced bad sleeping state, with a very short total sleep time and high sleep disruptions.

The noise detected (49.5±5.4dB) in poor sleepers in this study exceeded the WHO recommendations (about 35 dB at night) and was equivalent to the average noise levels detected in ICU over a 24-hour period [31, 32]. And this may be due to the use of equipment in ward after surgery. It has been shown that noise acts as a key factor for poor sleep and accounts for 11% to 24% of the total number of arousal during sleep [33]. Interestingly, both subjective perceived noise measured by RCSQ scale and objective noise detected by digital sound level meter were obtained in this study. Although both of them showed high correlation with sleep characteristics among early KTRs, only the self reported noise was found to be a statistically significant factor affecting sleep quality. Moreover, the recipients’ subjective sound perception (45.3±25.5 mm) was only detected at a moderate level, indicating that recipients might subjectively improve their noise tolerance. But the self-reported noise was still the main factor causing sleep disorder as shown in this study. Additionally, other factors should be also considered in this case such as using of sedative and analgesic drugs and the continuable ambient background noise (air conditioning, computer, ECG monitors, etc.) at night, both of them may weaken patient’s subjective noise perception.

Our findings emphasized the ability of the RCSQ to predict sleep efficiency. Although current research indicates that the RCSQ is a valid measurement of sleep perception, previous investigators have reported its mixed applications when compared to perceived and objective sleep latency [12]. In this study, we tried to understand the correlation between PSG and RCSQ among early KTRs. In contrast with the original research from RCSQ scale development, our study showed that most PSG measurements are significantly correlated with their corresponding RCSQ items, and the RCSQ total scores are significantly correlated with the number of awakenings, percent N2 and the total sleep time. Although In Richards’ research RCSQ only accounted for approximately one-third of the variance in sleep efficiency. In addition, we also noticed that the reported sleep quality and the overall sleep perception are more associated with the total sleep time, the percent of stage 2 sleep and the percent of REM in early KTRs. These conclusions are consistent with published literatures [22, 34, 35], indicating that postoperative patients need more full night’s sleep. While the total sleep time is reduced and the restorative slow sleep wave is disappeared due to the presence of various affecting factors. And this situation becomes more obvious in the first a few days postoperative [29].

Several limitations of this study should be considered when interpreting our results. First of all, we cannot make a generalized conclusion because of small number of examined samples. This is because most of KTRs were not familiar with PSG which resulted in loss of many participants with incomplete and discontinuous sleep data. Secondly, when analyzing the influencing factors for sleep, some variables such as dialysis duration and preoperative creatinine manifested a certain level of correlation, but they did not enter the regression equation. Evidence showed that the poor sleep quality is present in 88.5% of the hemodialysis patients and in 78.0% of the peritoneal dialysis patients [36]. The declined in sleep quality is associated with dialysis duration, 14.4% of patients display decreased sleep quality after one-year of dialysis [37]. Creatinine is an independent factor affecting the sleep quality [38]. But these variables did not produce a significant result may be due to small number of examined samples in this study.

## 5. Conclusions

The early KTRs show poor sleep quality as measured by both PSG and RCSQ. Sleep disruption always remains a substantial problem and is affected by self-reported noise in these patients. Because of the high consistency between subjective and objective measurements, it may encourage us to apply the RCSQ for clinical sleep monitoring in future daily practice.

## Supporting information

S1 Appendix Transplantation information of participants (n = 83). (DOCX)
