# Supplementary material for: What are the sleep characteristics among early kidney transplant recipients? An objective and subjective measurement from China
Source: PLoS One. 2022 Nov 22;17(11):e0277678. doi: 10.1371/journal.pone.0277678 (PMC9681072; doi:10.1371/journal.pone.0277678)
Supplement: S1 Appendix — (DOCX) [file pone.0277678.s001.docx]

**Transplantation information of participants (n=83)**

| **No** | **Operation time** | **No** | **Operation time** | **No** | **Operation time** |
| --- | --- | --- | --- | --- | --- |
| 1 | 2018.8.14 | 2 | 2018.8.14 | 3 | 2018.8.14 |
| 4 | 2018.8.14 | 5 | 2018.8.25 | 6 | 2018.8.27 |
| 7 | 2018.8.27 | 8 | 2018.9.02 | 9 | 2018.9.07 |
| 10 | 2018.9.12 | 11 | 2018.9.12 | 12 | 2018.10.3 |
| 13 | 2018.10.11 | 14 | 2019.10.11 | 15 | 2018.10.11 |
| 16 | 2018.10.11 | 17 | 2018.12.20 | 18 | 2018.12.20 |
| 19 | 2019.3.26 | 20 | 2019.3.26 | 21 | 2019.3.26 |
| 22 | 2019.3.29 | 23 | 2019.4.17 | 24 | 2019.4.17 |
| 25 | 2019.4.20 | 27 | 2019.5.01 | 28 | 2019.5.01 |
| 29 | 2019.5.05 | 30 | 2019.5.14 | 31 | 2019.5.14 |
| 32 | 2019.5.30 | 33 | 2019,5.30 | 34 | 2019.5.31 |
| 35 | 2019.5.31 | 36 | 2019.6.02 | 37 | 2019.6.03 |
| 38 | 3019.6.03 | 39 | 2019.6.03 | 40 | 2019.8.14 |
| 41 | 2019.8.15 | 42 | 2019.8.24 | 42 | 2019.8.24 |
| 43 | 2019.8.24 | 44 | 2019.8.24 | 45 | 2019.8.31 |
| 46 | 2019.9.03 | 47 | 2019.9.16 | 48 | 2019.9.16 |
| 49 | 2019.9.18 | 50 | 2019.9.22 | 51 | 2019.10.07 |
| 52 | 2019.10.07 | 53 | 2019.10.07 | 54 | 2019.10.07 |
| 55 | 2019.10.08 | 56 | 2019.10.15 | 57 | 2019.10.15 |
| 58 | 2019.10.17 | 59 | 2019.11.6 | 60 | 2019.11.6 |
| 61 | 2019.11.6 | 62 | 2019.11.20 | 63 | 2019.11.20 |
| 64 | 2019.11.23 | 65 | 2019.11.23 | 66 | 2019.12.04 |
| 67 | 2019.12.04 | 68 | 2020.11.07 | 69 | 2020.11.07 |
| 70 | 2020.11.17 | 71 | 2020.12.17 | 72 | 2020.12.19 |
| 73 | 2020.12.22 | 74 | 2020.12.22 | 75 | 2021.2.28 |
| 76 | 2021.2.28 | 77 | 2021.2.28 | 78 | 2021.2.28 |
| 79 | 2021.3.06 | 80 | 3031.3.06 | 81 | 2021.3.07 |
| 82 | 2021.3.11 | 83 | 2021.3.11 |  |  |
